# Body composition, physical activity, and quality of life in pediatric patients with inflammatory bowel disease on anti-TNF therapy—an observational follow-up study

**DOI:** 10.1038/s41430-022-01245-9

**Published:** 2022-12-07

**Authors:** Kriszta Katinka Boros, Gábor Veres, Orsolya Cseprekál, Hajnalka Krisztina Pintér, Éva Richter, Áron Cseh, Antal Dezsőfi-Gottl, András Arató, György Reusz, Dóra Dohos, Katalin Eszter Müller

**Affiliations:** 1 1st Department of Pediatrics, Semmelweis University, Budapest, Hungary; 2 Pediatrics Clinic University of Debrecen, Clinical Center ÁOK, DEKK, Debrecen, Hungary; 3 Department of Transplantation and Surgery, Semmelweis University, Budapest, Hungary; 4 Semmelweis University, School of Ph.D. studies, Budapest, Hungary; 5 Institute for Translational Medicine, University of Pécs, Pécs, Hungary; 6 Heim Pál National Pediatric Institute, Budapest, Hungary

**Keywords:** Paediatrics, Nutrition

## Abstract

**Background:**

Poor outcome of inflammatory bowel disease (IBD) is associated with malnutrition. Our aim was to compare body composition (BC) and physical activity (PA) between patients with IBD and healthy controls, and to assess the changes in BC, PA and health related quality of life (HRQoL) in children with IBD during anti-TNF therapy.

**Methods:**

32 children with IBD (21 with Crohn’s disease (CD), (age: 15.2 ± 2.6 years, 9 male) and 11 with ulcerative colitis (UC), (age: 16.4 ± 2.2 years, 5 male) participated in this prospective, observational follow up study conducted at Semmelweis University, Hungary. As control population, 307 children (age: 14.3 ± 2.1) (mean ± SD) were included. We assessed BC via bioelectric impedance, PA and HRQoL by questionnaires at initiation of anti-TNF therapy, and at two and six months later. The general linear model and Friedman test were applied to track changes in each variable.

**Results:**

During follow-up, the fat-free mass Z score of children with CD increased significantly (-0.3 vs 0.1, *p* = 0.04), while the BC of patients with UC did not change. PA of CD patients was lower at baseline compared to healthy controls (1.1 vs. 2.4), but by the end of the follow up the difference disappeared.

**Conclusions:**

The fat-free mass as well as PA of CD patients increased during the first six months of anti-TNF treatment. As malnutrition and inactivity affects children with IBD during an important physical and mental developmental period, encouraging them to engage in more physical activity, and monitoring nutritional status should be an important goal in patient care.

## Introduction

Inflammatory bowel diseases (IBD), including Crohn’s disease (CD) and ulcerative colitis (UC), have a significant negative effect on health-related quality of life (HRQoL) and physical activity (PA), furthermore, is it often associated with undernutrition [1, 2].

Malnutrition is a condition, when the imbalance of energy, protein, and other nutrients causing adverse effects on function, clinical outcome and body form (e.g. body composition (BC)), such as loss of lean mass or fat-free mass (FFM, body weight without fat mass) and / or skeletal muscle mass (SMM), even with normal body mass index (BMI) [3–5]. The loss of SMM increases the risk for sarcopenia, which is a predictor of adverse clinical outcomes, and is associated with a higher risk for relapse, need for surgery and reduced efficacy of biologicals in children with IBD [6]. Malnutrition and impaired BC may contribute to impaired HRQoL in patients with IBD [7]. In addition, PA is also associated with HRQoL and BC, as it was found to have a positive influence on HRQoL and lean body mass in patients with IBD [7–10].

Elevated TNF-α levels are known to be involved in skeletal muscle loss and growth impairment [11]. According to studies in adults, anti-TNF therapy may have a potential positive effect on sarcopenia by inhibiting inflammation and catabolism in skeletal muscles, however the literature is limited [12–15].

The BC, HRQoL, and PA triad seems to be related [7–10, 16, 17]. Though, changes in BC, PA, and HRQoL during biological therapy in pediatric IBD have not been studied. Only one cross-sectional study including patients with quiescent and mild disease assessed these factors and found significantly lower lean mass, grip strength, and PA compared to controls [17]. Therefore, our primary aim was to assess the changes in BC, HRQoL and PA of children with IBD initiating anti-TNF therapy and to compare BC and PA between patients with IBD and healthy controls. Our secondary aim was to analyse baseline characteristics of patients with or without risk of sarcopenia at the beginning of anti-TNF therapy and to follow the changes in BC.

## Materials and methods

From October 2016 to December 2018, patients with pediatric IBD starting anti-TNF therapy were recruited at the Ist Department of Pediatrics at Semmelweis University, Budapest, Hungary for assessment of BC, HRQoL, and PA in this prospective single-center, observational cohort study.

### Patients

Patients with IBD initiating anti-TNF therapy were consecutively included if they were between 10 and 19 years of age and signed an informed consent. Exclusion criteria were concomitant conditions affecting BC, PA, or HRQoL (e.g., autism spectrum disorder, edema, cirrhosis, hypoalbuminemia, hypersensitivity to infliximab, or other reasons leading to discontinuation of therapy, associated endocrine or chronic disorders (e.g., diabetes mellitus), known active malignancy, and lack of informed consent).

The diagnosis of IBD was based on the Porto criteria [18]. Anti-TNF therapy was indicated based on the administration criteria of the National Health Insurance Fund of Hungary and international guidelines [19, 20].

It is recommended to use population and device specific reference values for appropriate BC evaluation with BIA devices [21]. Therefore, 307 healthy children aged 10-18 years from local secondary schools were also included in this study to create reference values. Exclusion criteria for the control group were acute diseases, such as infections during the last 4 weeks before the evaluation, and chronic disorders or physical disabilities. In addition, controls also filled out the PA questionnaire. This way BC as well as PA could also be compared between children with IBD and controls.

### Procedure and measures

#### Study design

BC, HRQoL, PA, disease activity, and laboratory parameters were assessed at the start of anti-TNF therapy (measurement 0, M0), at the end of induction (M2), and at 6 months (M6).

#### Anthropometry and body composition

We measured height using a stadiometer and calculated BMI as weight [kg]/height[m2]. BMI, weight, and height were compared with the national longitudinal growth reference [22]. BC was assessed using bioelectrical impedance analysis with a multi-channel device at 5, 50, 250, 500, and 1000 kHz (InBody 720 (Biospace Co, Ltd, Seoul, Korea)). BC was measured before noon (8:00-12:00), after at least two hours fasting in minimal clothing, with abducted upper extremities (30°) without jewelry and watches. We extracted the FFM, SMM, and body fat mass (BFM) data from the device records.

#### Physical activity

To assess PA, two self-administered, 7-day recall questionnaires, the Canadian Physical Activity Questionnaire for Older Children (PAQ-C) and Adolescents (PAQ-A) were adapted and then applied. The questionnaire consists of 10 questions and provides an activity score between 1–5 (where 1 represents low activity and 5 represents high activity level) [23].

The questionnaire was completed by all patients at every measurement point. In addition, 204 children from the healthy control group completed the questionnaire. The PA of patients with IBD was compared to age-, sex-, and BMI-matched controls; the ratio of the cases and controls was 1:3.

#### Health related quality of life assessment

HRQoL was assessed using the disease-specific Canadian IMPACT-III Quality of Life questionnaire, adapted to Hungarian by Szabó et al. [24, 25]. It consists of six subscales (bowel symptoms, systemic symptoms, emotional functioning, social functioning, body image, and treatment) with 35 questions. Possible scores range from 35 to 175.

#### Patient-related parameters

Disease-specific data (e.g., disease location, laboratory parameters, and concomitant medical therapy) were obtained from medical records at the time of bioimpedance measurement. The Paris Classification was applied for the localization and disease phenotype [26]. Disease severity was estimated using the Pediatric Crohn’s Disease Activity Index (PCDAI) and Pediatric Ulcerative Colitis Activity Index (PUCAI) scores [27, 28]. Remission was defined as PCDAI ≤ 10 in the CD group, and response to therapy was defined as a ≥15-point reduction in PCDAI. In the UC group, the cut-off point of remission was PUCAI < 10 points, and response to therapy was defined as a decrease in PUCAI score ≥20 points.

#### Statistical analysis

Continuous variables are shown as mean ± SD in the tables. Height, weight, and BMI values were converted to age- and sex-specific standard deviation Z scores, using Hungarian population-based data (22).

Reference values for BC parameters (FFM, SMM, and BFM) were generated from the data of the healthy cohort group with the LMS (Lambda Mu and Sigma method), method using Statistica software. The LMS method summarizes the changing distribution by three curves representing the median (M), coefficient of variation ((S), ie, the ratio of the SD and mean) and skewness (L), transforming data to normality [29]. We used age, sex, and BMI-based Z scores to analyze BC data of IBD patients.

Baseline characteristics, anthropometric and BC parameters, HRQoL, PA, and their changes during the study period were analyzed. We compared these parameters between patients with CD and UC. In addition, we divided the patients into two groups based on the baseline SMM Z score. Patients were considered to have a risk of sarcopenia based on SMM Z score ≤-1 (Group A); these patients were compared to children (Group B) whose SMM Z score was >-1.

An independent *t* test was applied to analyze the difference in baseline characteristics between groups. The general linear model (GLM) was applied to track changes in each variable of BC, laboratory parameters, and disease activity indices throughout the study period. Pearson’s correlation coefficient was used for the correlation probes. The level of significance was set at *p* < 0.05. The distribution of the results of the HRQoL and PA questionnaires was non-parametric; thus, the Friedman test was performed. Post hoc analysis with Wilcoxon signed rank test was conducted with a Bonferroni correction, resulting in a significance level of *p* < 0.017. HRQoL and PA were compared between the groups using the Mann-Whitney U test. Questionnaires’ results are shown in median + IQR in figures. All analyses were performed using IBM SPSS Statistics for Windows, version 20 (Chicago, IL).

### Ethics

The study was approved by the Semmelweis University Institutional Committee for Research Ethics (SE TUKEB No: 215/2016).

## Results

### Baseline clinical and demographic characteristics

During the study period, thirty-five patients started biological therapy, three of whom were excluded due to autism spectrum disorder (*n* = 1), young age (younger than 10 years) (*n* = 1), and leg fracture (*n* = 1) (Supplementary Fig. 1). Altogether, 32 IBD patients, 21 CD (age: 15.2 ± 2.6 years (mean + SD), 9 male) and 11 UC patients (age: 16.4 ± 2.2 years, 5 male) were enrolled. Anti-TNF agents (adalimumab and infliximab) were administered according to the standard dosing protocols, except for four patients who needed intensification based on clinical judgment during the follow-up. There were no patients who stopped anti-TNF therapy during the study period.

Patients with CD had a significantly lower hemoglobin and a higher thrombocyte level, compared to UC group. The median time between diagnosis and starting anti-TNF therapy was 1.4 and 3 years in CD and UC group, respectively. Most of the CD patients had an ileocolonic (42.8%) or colonic (33.3%) disease. Among UC patients, most of the patients had left sided disease (81.8%). Concomitant therapy was azathioprine in 80% and 63% in patients with CD and UC, respectively, in addition, 19% and 36% of patients received systemic steroids. Further data on baseline demographics, clinical characteristics, biomedical markers, and current therapy are summarized in Table 1.

**Table 1:** Baseline demographic and clinical characteristics of patients with Crohn’s disease and ulcerative colitis.

Parameters	Crohn’s disease	Ulcerative colitis	*p*
Number of patients	21	11	*p* > 0.05
Age in years, mean+SD	15.2 ± 2.6	16.4 ± 2.2	*p* > 0.05
Gender, male % (n)	42% (9)	45% (51)	
Median time between diagnosis and starting anti-TNF therapy(year) [IQR]	1.4 [3.6]	3.0 [3.3]	*p* > 0.05
PCDAI/PUCAI (mean ± SD)	23.1 ± 14.0	27.3 ± 23.0	
CRP (mg/l, mean ± SD)	30.8 ± 43.8	14.1 ± 29.3	*p* > 0.05
Hemoglobin, g/dL, mean+SD	114.0 ± 19.8	129.6 ± 16.6	*p* = 0.03
Thrombocytes (G/L, mean ± SD)	451.0 ± 169.4	285.3 ± 120.9	*p* = 0.005
Albumin (g/L, mean+SD)	38.6 ± 5.8	42.4 ± 7.7	*p* > 0.05
Extent / Location of the disease			
L1	23.8% (5)		
L2	33.3% (7)		
L3	42.8% (9)		
L4	71.4% (15)		
E1		0	
E2		81.8% (9)	
E3		0	
E4		18.2% (2)	
Disease Behavior (B1, B2, B3)			
B1	14		
B2	5		
B3	2		
Medical treatment % (n)			
5-ASA	66% (14)	90 (10)	
AZA	80% (17)	63% (7)	
MTX	0	9% (1)	
Antibiotics	42% (9)	27% (3)	
Systemic corticosteroid	19% (4)	36% (4)	
Topical steroid	14% (3)	27/% (3)	

*PCDAI* Pediatric Crohn’s Disease Activity Index, *PUCAI* Pediatric Ulcerative Colitis Activity Index, *CRP* C-reactive protein, *L1* Ileal, *L2* Colonic, *L3* Ileocolonic, *L4* Upper gastrointestinal tract, *B1* Non-stricturing, non-penetrating, *B2* Stricturing, *B3* Penetrating, *E1* Ulcerative proctitis, *E2* left-sided UC (distal UC), *E3* extensive (hepatic flexure distally), *E4* pancolitis (proximal to hepatic flexure), *5-ASA* 5-aminosalicylic acid, *AZA* azathioprine, *MTX* methotrexate, *SD* standard deviation.

By the end of the induction period (M2), 58% of the patients with CD (10/17) and 37.5% of the patients with UC (3/8) achieved clinical remission. One CD patient and one UC patient responded to anti-TNF therapy without achieving clinical remission. After six months of anti-TNF therapy, 21% of the children with CD (3/14) and 45% of the patients with UC (5/11) still had an active disease (Supplementary Fig. 2).

### Body composition at baseline and during the first six months of anti-TNF therapy

At baseline, the mean BMI Z score was –1.1 of children the CD and -0.9 in the UC group (Table 2). Height and weight Z scores were not significantly different between the CD and UC groups. Patients with CD had a significantly lower FFM Z score compared to the UC group (–0.4 vs 0.5 *p* = 0.04); meanwhile, the mean BFM Z score was 1.0 in both groups. There was a high correlation in the UC group between albumin and BFM Z scores (R = 0.6, *p* = 0.03), and hemoglobin and BFM Z scores (R = 0.7, *p* = 0.02).

**Table 2:** Baseline body composition parameters of patients with Crohn’s disease and ulcerative colitis.

Parameters	Crohn’s disease	Ulcerative colitis	*p*
Height Z score	–0.2 ± 1.2	0.6 ± 1.1	*p* > 0.05
Weight Z score	–1.0 ± 0.8	–0.6 ± 0.7	*p* > 0.05
BMI Z score	–1.1 ± 0.7	–0.9 ± 0.7	*p* > 0.05
FFM Z score	–0.4 ± 1.1	0.5 ± 1.3	*p* = 0.04
SMM Z score	–0.5 ± 1.1	0.4 ± 1.3	*p* > 0.05
BFM Z score	1.0 ± 1.2	1.0 ± 1.3	*p* > 0.05

*BMI* body mass index, *FFM* fat free mass, *SMM* skeletal muscle mass, *BFM* body fat mass.

**Fig. 1:**
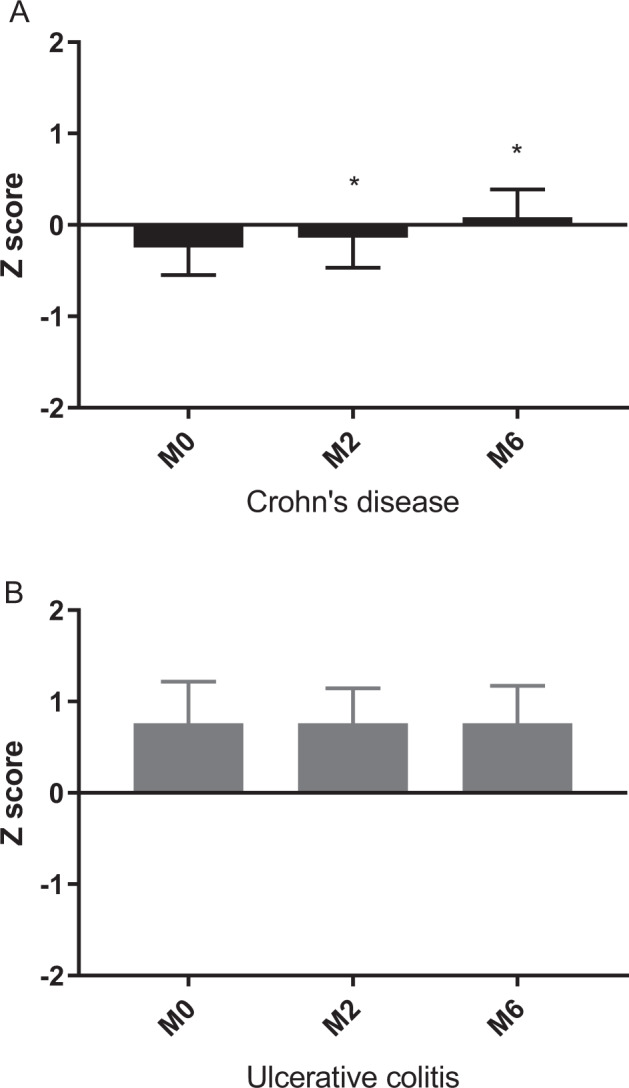
Fat free mass Z score changes in patients. **A** CD Crohn’s disease, M0 measurement 0, M2 measurement 2, M6 measurement 6, *: vs. M0, *p* < 0.05. **B** UC ulcerative colitis, M0 measurement 0, M2 measurement 2, M6 measurement 6.

During the study period, mean height, weight, and BMI Z scores did not change in children with CD or UC (Supplementary Table 1). However, mean FFM Z score increased significantly in the CD group (M0: –0.3 ± 1.2, M2: –0.1 ± 1.1, M6: 0.1 ± 1.2, *p* < 0.05) (Fig. 1A; Supplementary Table 1). The BC parameters in the UC group did not change during the first six months of anti-TNF therapy (Fig. 1B; Supplementary Table 1).

### HRQoL and physical activity during the first six months of anti-TNF therapy

At baseline, the median IMPACT-III score was 128.5 (pc 25, 75: 111.5; 137.8) in patients with CD and 109 (pc 25, 75: 83; 129) in patients with UC. HRQoL did not change significantly (CD: M0, 128.5; M6, 144; UC: M0, 109; M6, 116) Supplementary Table 2).

**Fig. 2:**
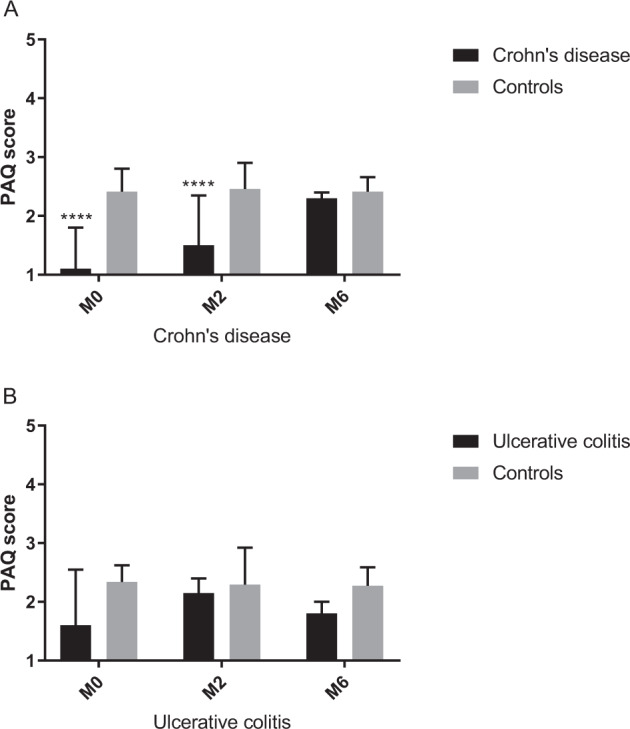
Physical activity of patients compared to age, sex and BMI Z score adjusted controls. **A** PAQ Physical activity questionnaire, M0 Measurement 0, M2 measurement 2, M6 measurement 6; ****: vs. Controls, *p* < 0.00005. **B** PAQ Physical activity questionnaire, M0 Measurement 0, M2 measurement 2, M6 measurement 6.

Comparing PA scores to age, sex, and corresponding BMI-adjusted controls, PA was lower in the CD group at baseline (CD: 1.1 vs. controls: 2.4), and the difference disappeared by M6 (2.3 vs. 2.4) (Fig. 2A).

In the UC group, PA was comparable to the control group at baseline, and it did not change throughout the study period (M0: 1.6 vs. 2.3, M6: 1.8 vs. 2.2) (Fig. 2B).

IMPACT III and PAQ scores showed a correlation at M0 in patients with CD (r = 0.73, *p* = 0.007).

### Subgroup analysis: comparison of children with or without risk of sarcopenia

**Fig. 3:**
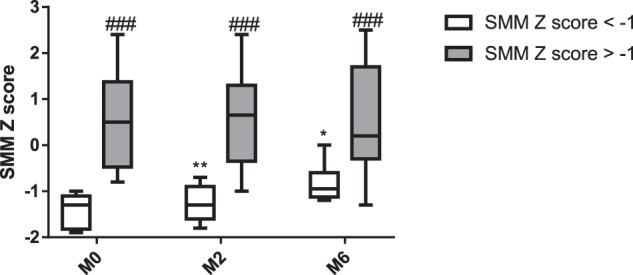
Changes of skeletal muscle mass Z score in children with or without risk of sarcopenia during the study period. SMM skeletal muscle mass, M0 measurement 0, M2 measurement 2, M6 measurement 6; *: vs. M0, *p* < 0.05; **:vs. M0, *p* < 0.005; ###: vs. the equivalent parameter in SMM Z score < -1, *p* < 0.0005.

As a subgroup analysis, patients were divided into two groups based on the baseline SMM Z score (Supplementary Table 3). Patients with risk of sarcopenia were significantly younger and shorter than patients without risk of sarcopenia (Group A vs. Group B, age: 13.9 ± 2.8 years vs. 16.4 ± 1.9 years, *p* < 0.05; height Z score: 0.7 ± 1.1 vs. 0.4 ± 1.0, *p* < 0.05, respectively) at the initiation of anti-TNF therapy. There was no significant difference in disease duration, activity indices, or any of the laboratory parameters between the two groups. At M2 and M6, the difference in height Z score disappeared between the two groups (Group A vs. Group B; M2: -0.8 ± 1.3 vs. 0.4 ± 1.0, M6: -0.4 ± 1.2 vs. 0.4 ± 1.1). The mean SMM Z score increased significantly in Group A; however, patients in Group A still had a significantly lower SMM Z score at M2 and M6 than children in Group B. SMM Z score did not change during the study period in Group B (Fig. 3 Supplementary Table 4). There was no difference in the HRQoL and PAQ between the two groups.

## Discussion

In this prospective observational study, we monitored BC, PA, and HRQoL in 32 patients with pediatric IBD during the first six months of anti-TNF treatment. We found that the mean FFM Z score increased significantly in patients with CD during the follow-up, but not in patients with UC. The PA of CD patients was lower than that of healthy controls but became comparable by the end of the study period. We also found that patients with risk of sarcopenia were younger and shorter compared to patients whose baseline SMM Z score was above –1.

The importance of nutritional status and BC has received increased attention in the past few decades due to the recognition of the association between malnutrition, sarcopenia, and poor prognosis. However, only one cross-sectional study evaluated BC, PA, and HRQoL of children with IBD in parallel [17].

In our study, patients with CD exhibited features of sarcopenia based on normal fat stores but decreased FFM at baseline. During anti-TNF therapy, an increasing FFM Z-score was observed among patients with CD which is in accordance with previous studies [30–32]. According to these studies, BC deficits in CD can be a consequence of immune-mediated mechanisms affecting growth hormone metabolism, which release during anti-TNF therapy [31, 32].

The mean FFM Z score was higher in the UC group at baseline than in the CD group, reflecting that malnutrition and loss of lean mass are less frequent in UC [33–37]. The previous data in pediatric population are controversial. Boot et al. reported similar results with us, while other studies showed no difference in protein-related compartments between IBD subgroups [38–40]. Meanwhile, Hill et al. found lower body cell mass Z scores, while BMI Z scores were comparable with healthy children, suggesting reduction of FFM in patients with UC [41].

We did not detect any significant changes in BC parameters in patients with UC, however they did not have deficits in BC parameters at baseline. As systemic inflammation is also a part of the pathogenesis of UC, and could affect BC as well [42]. Csontos et al. showed smaller change of muscle parameters in adult patients with UC than in patients with CD during anti-TNF therapy [14]. Best to our knowledge, BC has not yet been reported in pediatric UC patients receiving anti-TNF therapy.

Previous studies have reported increasing HRQoL in children during anti-TNF therapy [25, 43]. Controversy, we did not find improvement in HRQoL though there was a tendency to increase. It is of note that the baseline IMPACT-III score values of our patients were higher and PCDAI values were lower at the time of initiation of anti-TNF therapy than in earlier studies [25]. This probably reflects the upcoming practice of early step-down treatment in high-risk patients. The higher baseline IMPACT-III and lower PCDAI scores reflect that the anti-TNF was initiated at an earlier stage of the disease, so we could “maintain” the HRQoL of our patients with anti-TNF therapy.

PA was lower in patients with IBD than in healthy controls at baseline. Data on the association between anti-TNF therapy and PA from earlier studies are limited and conflicting [13, 44]. The lack of increase in PA in our study may be partly due to parental overprotective behavior and ongoing disease activity. However, the increase in FFM without a significant change in PA may suggest the role of anti-TNF via mucosal healing and blockade of lipolytic and proteolytic effects of inflammatory cytokines.

Our secondary aim was to compare patients with risk (SMM Z score ≤–1) and without risk (SMM Z score > -1) of sarcopenia. While in adults, sarcopenia is defined as decreased muscle mass and muscle strength [45], there is no consensus in the definition of sarcopenia in pediatric population [6]. In our study, we considered children with a lower SMM Z score (baseline SMM Z score ≤–1) to have a risk of sarcopenia. Comparing the baseline characteristics of children with and without risk of sarcopenia, we found, that patients with a risk of sarcopenia were younger and shorter than those with SMM Z score of > -1, and they had a significant increase in SMM Z scores, in contrast to patients, without the risk of sarcopenia. This finding is probably due to the high growth velocity in early puberty, or maybe because of the greater impact of anti-TNF agents in this population [46]. Both of them calls attention to the vulnerability of children in early pubertal age. Walters et al. also observed that infliximab was associated with an increased height Z score in patients with early puberty [47]. These results suggest that the risk of impaired height and BC may be associated with younger age.

Our study had several strengths and limitations. The low number of cases, the short follow-up period, and the lack of information about nutritional intake may limit the general implications of the study. Furthermore, evaluation of changes in functional muscle parameters (grip strength) could complete our results. However, this is a prospective observational follow-up study assessing the “functioning” of our patients during anti-TNF therapy via evaluation of PA and HRQoL besides the nutritional status.

In addition, only a few data are available on the BC of pediatric UC patients, particularly, those undergoing anti-TNF therapy.

## Conclusion

We observed changes of FFM Z scores in children with CD during anti-TNF therapy, while weight and BMI did not change, which highlights that weight and BMI are not enough sensitive markers of nutritional status. We did not detect significant improvement in HRQoL and PA suggesting that patients need more time for functional recovery. The lower age of the patients with lower SMM Z scores draws attention to the vulnerability of younger patients. Malnutrition and physical inactivity affects children with IBD during an important physical and mental developmental period. Encouraging them to engage in more physical activity, and monitoring nutritional status should be an essential goal in patient care.

## Supplementary information

Supplemetary material

## Data Availability

The data underlying this article will be shared upon reasonable request from the corresponding author.
