# Supplementary material for: Body composition, physical activity, and quality of life in pediatric patients with inflammatory bowel disease on anti-TNF therapy—an observational follow-up study
Source: Eur J Clin Nutr. 2022 Dec 7;77(3):380–5. doi: 10.1038/s41430-022-01245-9 (PMC10017513; doi:10.1038/s41430-022-01245-9)
Supplement: Supplementary file 1 — Supplemetary material [file 41430_2022_1245_MOESM1_ESM.docx]

**Electronic Supplementary Material**

**Title: Body composition, physical activity, and quality of life in pediatric patients with inflammatory bowel disease on anti-TNF therapy – an observational follow-up study**

**Author:** Kriszta Katinka Boros ^1^*, Gábor Veres † ^2^, Orsolya Cseprekál ^3^, Hajnalka Pintér ^4^, Éva Richter ^1^, Áron Cseh ^1^, Antal Dezsőfi-Gottl ^1^, András Arató ^1^, György Reusz ^1^, Dóra Dohos ^5^, and Katalin Eszter Müller ^5,6^

**Supplementry table 1.** Comparison of body composition parameters in patients with Crohn’s disease and ulcerative colitis at M0, M2 and M6.

**Supplementary Table 2.** Health related quality of life and physical activity in patients with Crohn’s disease and ulcerative colitis during the study period

**Supplementary Table 3.** Patients with skeletal muscle mass score ≤ -1(Group A) compared to patients > -1 SMM Z score (Group B)

**Supplementary Table 4.** Comparison of body composition (BC) parameters in patients with SSM Z score ≤ -1 (Group A) and with SMM Z score > -1 (Group B)

**Supplementary Figure 1.** Flow chart

**Supplementary Figure 2.** Remission rate in patients with Crohn’s disease and ulcerative colitis during the study period.

**Supplementary Table 1. Comparison of body composition parameters in patients with Crohn’s disease and ulcerative colitis at M0, M2 and M6.**

|  | Crohn’s disease | | | Ulcerative colitis | | |
| --- | --- | --- | --- | --- | --- | --- |
|  | M0 | M2 | M6 | M0 | M2 | M6 |
|  | n=21 | n=17 | n=14 | n=11 | n=8 | n=11 |
| Height Z score  mean±SD | -0.1±1.3 | -0.1±1.3 | -0.1±1.2 | 0.9±1.1 | 0.8±1.0& | 0.8±1.0 |
| Weight Z score  mean±SD | -0.7±0.7 | -0.6±0.6 | -0.6±0.8 | -0.4±0.7 | -0.3±0.8 | -0.5±0.9 |
| BMI Z score  mean±SD | -0.8±0.5 | -0.7±0.4 | -0.6±0.5 | -0.9±0.7 | -0.7±0.6 | -0.9±0.8 |
| FFM Z score  mean±SD | -0.3±1.2 | -0.1±1.1* | 0.1±1.2* | 0.8±1.3 | 0.8±1.1& | 0.8±1.1 |
| SMM Z score  mean±SD | -0.3±1.1 | -0.2±1.1 | 0.0±1.2 | 0.7±1.3 | 0.7±1.1 | 0.7±1.2 |
| BFM Z score  mean±SD | 1.0±1.3 | 0.9±1.0 | 0.9±1.1 | 0.9±1.3 | 1.0±0.9 | 0.8±1.4 |

SD: standard deviation; BMI: body mass index; FFM: fat free mass; SMM: skeletal muscle mass; BFM: body fat mass; M0: measurement 0, M2: measurement 2, M6: measurement 6; *: vs. M0 p<0.05; & vs: the equivalent parameter in Crohn’s disease group, p<0.05

**Supplementary Table 2.** **Health related quality of life and physical activity in patients with Crohn’s disease and ulcerative colitis during the study period**

.

|  | Crohn’s disease (n=21) | | | Ulcerative colitis (n=11) | | |
| --- | --- | --- | --- | --- | --- | --- |
|  | M0 | M2 | M6 | M0 | M2 | M6 |
|  | Median (pc25,75) | Median (pc25,75) | Median (pc25,75) | Median (pc25,75) | Median (pc25,75) | Median (pc25,75) |
| Total IMPACT-III | 128.5 (111.5, 137.8) | 137.5 (125.3, 146.3) | 144  (126.8, 154.5) | 109 (83, 129) | 116 (94, 140) | 116 (95, 145) |
| (35-175) |  |  |  |  |  |  |
| PAQ  (1-5) | 1.1 (1.0, 1.8) | 1.5 (1.0, 2.3) | 2.3 (1.5, 2.4) | 1.6 (1.0, 2.5) | 2.15 (1.6, 2.4) | 1.8 (1.0, 2.0) |

PAQ: physical activity questionnaire; M0: measurement 0, M2: measurement 2, M6: measurement 6

**Supplementary Table 3. Patients with skeletal muscle mass score ≤ -1(Group A) compared to patients > -1 SMM Z score (Group B)**

| **Parameters** | **Group A** | **Group B** | **p** |
| --- | --- | --- | --- |
| Number of patients | 10 | 22 |  |
| Age in years, mean+SD | 13.9±2.8 | 16.4±1.9 | **p=0.02** |
| Gender, male % (n) | 20% (2/10) | 54.5% (12/22) |  |
| CD patients, % (n) | 80% (8/10) | 59% (13/22) |  |
| PCDAI (mean+SD) | 25.9±16.3 | 21.4±12.8 | p>0.05 |
| PUCAI (mean+SD) | 17.5±3.5 | 29.4±25 | p>0.05 |
| Mean disease duration (year) [IQR] | 3.0 [4.9] | 2.7 [2.5] | p>0.05 |
| CRP (mg/L, mean ± SD) | 36.2±60.4 | 19.8±24.6 | p>0.05 |
| Haemoglobin, g/dL, mean+SD | 115.3±24.9 | 121.1±17.4 | p>0.05 |
| Thrombocytes (G/L, mean ± SD) | 379.6±100.7 | 403.8±200.6 | p>0.05 |
| Albumin g/L, mean+SD | 39.0±7.9 | 40.3±6.0 | p>0.05 |
| Extent / Location of the disease |  |  |  |
| L1 | 5 |  |  |
| L2 | 7 |  |  |
| L3 | 9 |  |  |
| L4 | 15 |  |  |
| E1 |  | 0 |  |
| E2 |  | 9 |  |
| E3 |  | 0 |  |
| E4 |  | 2 |  |
| Disease Behaviour (B1, B2, B3) |  |  |  |
| B1 | 14 |  |  |
| B2 | 5 |  |  |
| B3 | 2 |  |  |

PCDAI: Pediatric Crohn’s Disease Activity Index, PUCAI: Pediatric Ulcerative Colitis Activity Index, CRP: C-reactive protein, L1: Ileal, L2: Colonic, L3: Ileocolonic, L4: Upper gastrointestinal tract, B1: Nonstricturing, nonpenetrating, B2: Stricturing, B3: Penetrating, E1: Ulcerative proctitis, E2: Left-sided UC (distal UC), E3: Extensive (hepatic flexure distally), E4: Pancolitis (proximal to hepatic flexure), 5-ASA: 5-aminosalicylic acid, AZA: Azathioprine, MTX: methotrexate; SD: standard deviation

**Supplementary Table 4. Comparison of body composition (BC) parameters in patients with SSM Z score ≤ -1 (Group A) and with SMM Z score > -1 (Group B)**

|  | Group A | | | Group B | | |
| --- | --- | --- | --- | --- | --- | --- |
|  | M0 | M2 | M6 | M0 | M2 | M6 |
|  | n=10 | n=7 | n=6 | n=22 | n=18 | n=19 |
| Height Z score  mean±SD | -0.7±1.1 | -0.8±1.3 | -0.4±1.2 | 0.4±1.0& | 0.4±1.0 | 0.4±1.1 |
| Weight Z score  mean±SD | -1.2±1.1 | -1.2±1.1 | -0.4±1.2 | -0.7±1.1 | -0.5±0.6 | -0.6±0.7 |
| BMI Z score  mean±SD | -1.0±1.0 | -1.0±1.1 | -0.3±0.7 | -1.0±0.6 | -0.8±0.4 | -0.9±0.5 |
| FFM Z score  mean±SD | -1.4±0.4 | -1.2±0.4 | -0.8±0.4 | 0.5±1.0 | 0.6±1.0 | 0.6±1.1 |
| SMM Z score  mean±SD | -1.4±0.4 | -1.3±0.4** | -0.8±0.4* | 0.4±1.0 | 0.5±1.0 | 0.6±1.1 |
| BFM Z score  mean±SD | 0.9±1.2 | 0.9±1.1 | 0.7±0.8 | 1.1±1.1 | 1.1±0.9 | 1.0±1.3 |

CD, Crohn’s disease; UC, ulcerative colitis; BMI, body mass index; FFM, fat free mass; SMM, skeletal muscle mass; BFM, body fat mass; M0: measurement 0, M2: measurement 2, M6: measurement 6; & vs: the equivalent parameter in SMM ≤ -1 group, p<0.05

**FIGURES**

**Supplementary Figure 1. Flow chart**

Patients number at M2: n=25

- CD n=17

- UC n=8

Patiens number during the study period: n=35

- CD n=22

- UC n=13

Patiens, meeting the inclusion criteria: n=32

- CD n=21

- UC n=11

Patients number at M6: n=25

- CD n=14

- UC n= 11

Excluded : 3 patients

- - Autism spectrum disorder
- - Age under 10 ys
- - Leg fracture

**Supplementary Figure 2. Remission rate in patients with Crohn’s disease and ulcerative colitis during the study period.**

M0: measurement 0, M2: measurement 2, M6: measurement 6
